# Use of the J774A.1 Cell Line as a Model in the In Vitro Study of Extracellular Vesicle Secretion from Histiocytic Sarcoma in Patients with Bacterial Co-Infections

**DOI:** 10.3390/ijms27114949

**Published:** 2026-05-29

**Authors:** Francisco Sierra-López, Susana Bernardo-Hernández, Lidia Baylón-Pacheco, Verónica Ivonne Hernández-Ramírez, Vanessa Iglesias-Vázquez, Rosa Martha Morales-López, Juan Carlos Fernández Hernández, Gustavo Acosta Altamirano, Patricia Talamás-Rohana, José Luis Rosales-Encina, Mónica Sierra-Martínez

**Affiliations:** 1 Department of Infectomics and Molecular Pathogenesis, Center for Research and Advanced Studies, Av. IPN 2508, Zacatenco, Mexico City 07360, Mexico; luck19861990@gmail.com (F.S.-L.); lbaylon@cinvestav.mx (L.B.-P.); arturomvi@hotmail.com (V.I.H.-R.); ptr@cinvestav.mx (P.T.-R.); 2 Health Research Unit, Hospital Regional de Alta Especialidad de Ixtapaluca, Servicios de Salud del Instituto Mexicano del Seguro Social para el Bienestar (IMSS-BIENESTAR), Carr Mex-Puebla Km 34.5 col., Zoquiapan, Mexico City 56530, Mexico; susanamex2007@hotmail.com (S.B.-H.); vanessa.iglesias2711@gmail.com (V.I.-V.); juan.fernandez@salud.gob.mx (J.C.F.H.); 3 Pathology Service, Hospital Regional de Alta Especialidad de Ixtapaluca, Servicios de Salud del Instituto Mexicano del Seguro Social para el Bienestar (IMSS-BIENESTAR), Carr Mex-Puebla Km 34.5 col., Zoquiapan, Mexico City 56530, Mexico; dramorales.pqc@gmail.com; 4 Hospital General de México “Dr. Eduardo Liceaga”, Eje 2A Sur (Dr. Balmis) No. 148, Cuauhtémoc, Doctores, CDMX, Mexico City 06726, Mexico; gustavo.acostaa@salud.gob.mx

**Keywords:** cancer, extracellular vesicles (EVs), histiocytic sarcoma (HS), macrophage, polydisperse EVs (PEVs)

## Abstract

Histiocytic sarcoma (HS) is an aggressive hematological malignancy whose transformed cells exhibit morphological and immunophenotypic characteristics similar to macrophages, and arises de novo or as part of a clonal ‘evolution’ of other pre-existing hematological neoplasms. This study investigates the potential use of the J774A.1 cell line (a cell line derived from murine tumor cells, commonly used in macrophage research) as a research model to study the role of polydisperse extracellular vesicles (PEVs) secreted by the HS cells, considering that bacterial infections are common in patients with cancer, including HS. The influences of bacterial components on tumor progression are still not fully understood. We stimulated the J774A.1 cell line in vitro with a fraction of *E. coli*, and our results show that the bacterial stimulation increases the secretion of PEVs by these cells. Comparative results of J774A.1 cells with PEVs using confocal and scanning electron microscopy with micrographic reports of HS histological slides (from several cited mammal species, including humans) suggest a possible relationship of large PEVs with marks, footprints, or traces of possible large PEVs disrupted in the HS of these reports. A subsequent proteomic analysis of these PEVs revealed a diverse subcellular origin of their components, such as proteins including: Triosephosphate isomerase (TPI), Heat shock cognate 71 kDa, Apolipoprotein A-1, Rho GDP-dissociation inhibitor 1, GAPDH, Galectin, Moesin, globular Actin, and Annexin. These results highlight the importance of studying the interplay between HS, other hematological cancers, and bacterial infections to better understand the progression of this cancer, identify new therapeutic targets, and emphasize the importance of preventing bacterial infections in cancer patients. Furthermore, the results demonstrate the potential use of the stimulated J774A.1 cell line for research on HS-related PEVs.

## 1. Introduction

Histiocytic sarcoma (HS) is an aggressive hematological malignancy whose cells exhibit morphological and immunophenotypic characteristics similar to mature histiocytes or tissue macrophages. It can arise de novo [1,2], or as part of a clonal “evolution” of other pre-existing hematological neoplasms [3]. For decades, it has been considered to represent less than 1% of all diagnosed hematological malignancies [4,5]. Statistics on HS detection are influenced by a probable combination of low biological incidence and a high rate of misdiagnosis (or underdiagnosis) [4,6]. This requires the exclusion of other diseases and extensive pathological and immunophenotypic analyses to ensure that the malignant cells possess the features of mature histiocytes (expressing markers such as CD68 and CD163) and lack the specific markers of other cell lineages (T cells, B cells, etc.) [1,5]. In the clinical course of HS, a wide range of organs can be affected, including soft tissues, lymph nodes, skin, the respiratory system, the gastrointestinal tract, and the spleen [2].

For HS research, there are various cell models that can represent different characteristics of HS, including aspects of cell physiology. The murine macrophage cell line J774A.1 is a subclone derived from the J774 reticulum cell sarcoma model, isolated from a mouse with this neoplasm [7,8,9]. This cell line is also commonly used for macrophage research [10]. Cells from the J774A.1 line is capable of secreting extracellular vesicles (EVs) constitutively and in response to stimuli (such as infection), which have been shown to have biological effects, such as modulating the immune response or reducing the bacterial load in infection models [11,12]. The variety of EVs that the J774A.1 line can secrete under stimulation is very diverse, ranging from small exosomes to EVs that reach sizes that allow them to be observed by a light microscope [13].

EVs are nano- and micro-particles enclosed by at least one lipid bilayer, limiting them from the outside. They are released by a cell, either originating in multivesicular bodies (exosomes) inside cells or by evagination of the plasma membrane (microvesicles, ectosomes). These particles are structures that carry a diverse bioactive cargo (proteins, lipids, DNA, RNA) [14,15]. In the context of oncology, EVs have emerged as key players in intercellular communication, with their cargo influencing the immune system, metastasis, response to treatment, and modulating the tumor microenvironment [16,17,18]. EVs have been associated with actively promoting the formation of pre-metastatic niches [19,20]. Due to their involvement in the communication, progression, and evolution of cancers of origin, EVs have come to be known as oncosomes (similar in size to exosomes) and large oncosomes (large EVs) [21].

The above-mentioned characteristics of the J774A.1 cell line makes it useful for studying the secretion and function of EVs as a model oriented toward the histiocytic–sarcomatous origin of these cells. Research into EVs in histiocytic cancer is relevant because, as reported in recent years, EVs are involved in the development of different types of cancer and could be involved in the pathogenesis and progression of HS, which in turn could show structural and functional similarities with EVs of cancerous origin and reflect the involvement of cell-to-cell communication in the disease.

## 2. Results

### 2.1. J774A.1 PEVs Stimulation, Secretion, and Isolation

**Figure 1:**
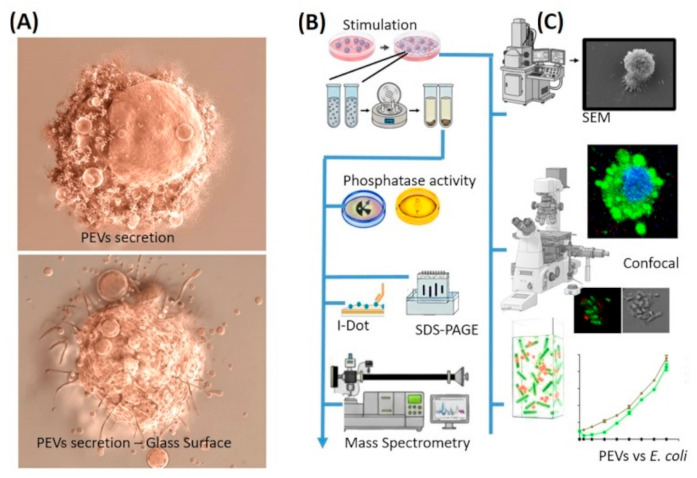
Schematic representation of J774A.1 PEVs secretion and multimodal analysis. (**A**) Phase-contrast images processed and digitally rendered for coloring, contrast enhancement, and visualization of PEVs secreted by a single J774A.1 cell stimulated with the SBMF of *E*. *coli* bacteria. (**A**) z-positions were chosen to focus on the cell (upper panel) and another view closer to the glass surface (bottom panel) to evidence the presence of the secreted vesicles by confocal microscopy. (**B**) Analysis strategies of PEVs obtained by centrifugation with or without ZnSO_4_. (**C**) Analysis process of PEVs using microscopy techniques and PEVs—EGFP (Enhanced green fluorescent protein) *E. coli* bacteria interactions. SEM = Scanning electron microscopy.

The J774A.1 cell line, derived from a mouse sarcoma (reticular cell sarcoma), is transformed and immortalized and expresses proteins characteristic of macrophages; therefore, it can be considered a direct analog of human HS. To obtain a wide variety of polydisperse extracellular vesicles (PEVs) secreted by J774A.1 cells (Figure 1A), the cells were first stimulated with a ‘Soluble Bacterial Membrane Fraction’ (SBMF) from the outer membrane of *Escherichia coli*, a fraction that is soluble in SDS that, after dialysis against water, was immobilized on culture surfaces using human fibronectin (FN). Once PEV secretion was induced, these vesicles were further analyzed using various microscopy strategies, as well as their biological activity, and in silico analysis of their protein composition (Figure 1B,C).

**Figure 2:**
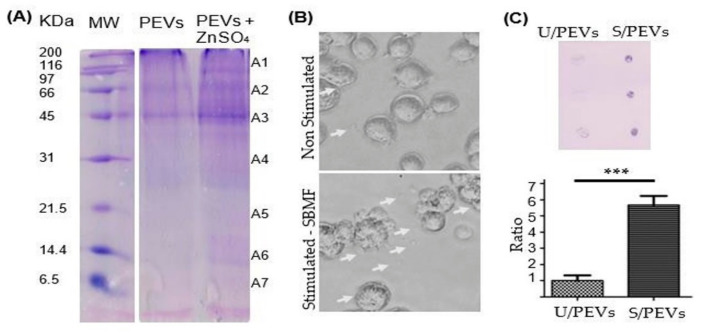
Stimulation of the secretion of J774A.1 PEVs linked to histiocytic sarcoma. (**A**) SDS-PAGE (12%) of PEVs obtained by centrifugation without ZnSO_4_ (PEVs) or with ZnSO_4_ (PEVs + ZnSO_4_) from the same proportion of J774A.1 cells stimulated with *E. coli* SBMF stabilized with FN. The protein profile (A1–A7) is similar to that reported in Sierra-López et al., 2025 for these PEVs [13]. (**B**) Images obtained from J774A.1 cells unstimulated or stimulated with the SBMF-FN (20× inverted microscope), where the arrows point to particles similar to large PEVs. (**C**) Representative immunodot blots of supernatants with secreted PEVs were analyzed using a mouse polyclonal anti-LMW-PTP (recognizes mouse and human LMW-PTPs) and an anti-mouse IgG AP as secondary antibody. PEVs from supernatants of unstimulated (U/PEVs) or stimulated cells (S/PEVs). All cells were stimulated for 2 h. A statistically significant difference was observed in the increase in detection in the stimulated supernatant. One-way ANOVA post hoc Bonferroni test, *** *p* < 0.0001 as significant, *n* = 5. MW = molecular weight marker; PEVs = polydisperse extracellular vesicles; UPEVs = unstimulated PEVs; SPEVs = stimulated PEVs.

Figure 2A shows an SDS-PAGE result comparing two strategies for PEVs obtained from 2 h stimulated cell culture supernatants. Both strategies used a centrifugation process, differing only in the addition of ZnSO_4_ to increase the PEVs precipitation. Generally, the strategy that included ZnSO_4_ (PEVs+ ZnSO_4_, classified as ‘Salting out’) resulted in a larger pellet. This increased recovery efficiency was also reflected in the gel analysis: when both pellets were dissolved in the same volume, and the same amount (µL) was loaded onto the gel, the sample obtained with ZnSO_4_ showed a greater intensity in its profile, indicating a higher concentration of PEVs. The banding patterns were similar under both conditions, with high-molecular-weight bands (approximately 97–200 kDa) standing out in the area labeled A1. In the gel, bands approximately in the A3 area (approximately 45 kDa), A2 (close to 66 kDa), and subsequently those below A4, A5 (between 14.4 and 31 kDa), and close to A6, A7 (close to 6.5 kDa) are prominent.

Figure 2B shows light microscopy images of J774A.1 cells that released particles resembling large EVs (white arrows), suggesting the release of PEVs, which are rarely found in unstimulated cultures. Figure 2C, an immunodot blot, confirms this finding by increasing the detection of Low Molecular Weight Protein Tyrosine Phosphatases (LMW-PTPs) in supernatants, whose antigens are loaded in J774A.1 PEVs, also showing statistical significance in line with what was observed in the cultures. A relationship was obtained between a higher quantity of PEVs and greater detection of LMW-PTPs in supernatants.

### 2.2. J774A.1 PEVs Scanning Electron Microscopy (SEM)

**Figure 3:**
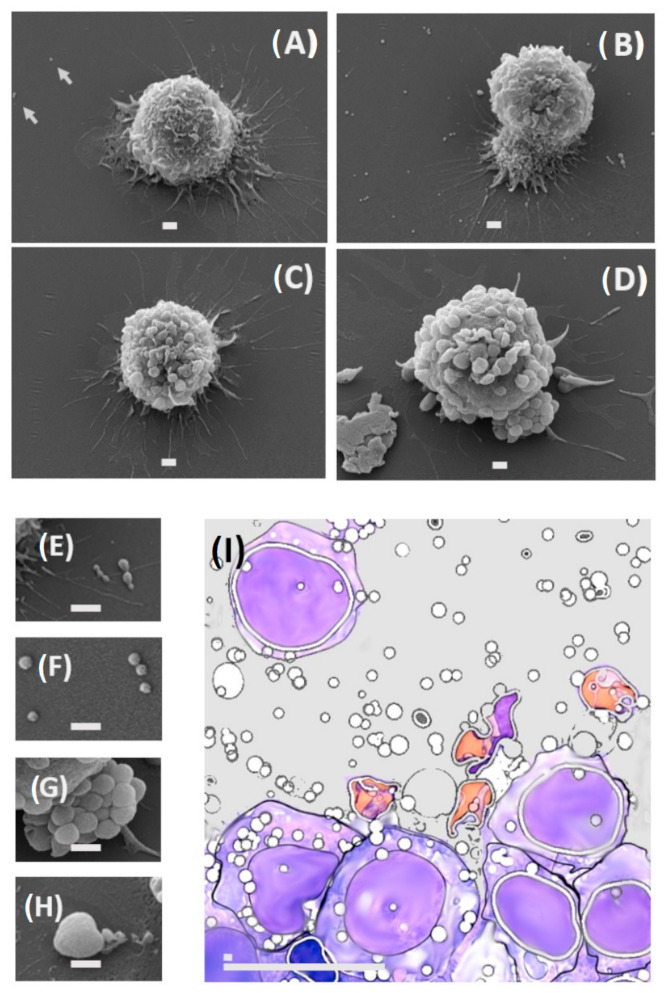
Scanning electron microscopy of J774A.1 cells secreting PEVs. The J774A.1 cell line was stimulated with a small amount of *E. coli* SBMF stabilized with FN, distributed as a substrate on the cell culture surface, for 2 h of interaction. (**A**) The results occasionally show cells with a normal or unstimulated appearance; this result suggests that those cells that did not come into contact with the substrate showed fewer PEVs, indicated by arrows. (**B**–**D**) Cells that came into contact with the substrate generally changed their morphology and exhibited PEVs. (**E**–**H**) Stimulated J774A.1 PEVs. Scale bar (**A**–**H**) 1 µm. (**I**) Schematic image illustrates a biopsy of a submandibular lymph node, erased by metastatic histiocytic sarcoma, large scale 20 µm and short scale 1 µm, based on Purzycka et al. 2020 [22].

Once the J774A.1 samples stimulated with SBMF-FN on glass were obtained, and the morphology of the PEVs was confirmed. In Figure 3A, the results occasionally show cells with a normal or unstimulated appearance. Figure 3B–D shows some characteristics acquired by the cells that interacted with the SBMF-FN substrate and actively secreted PEVs. PEVs tend to be spherical. Furthermore, during the cultures, PEVs were apparently anchored or adhered to the surface of the cell culture, and possibly were the substrate. In the SEM microphotographs, PEVs can be seen in rows (Figure 3E–H). The range of sizes of the PEVs shown in the SEM was from small ones close to 100 nm (Figure 3E) to larger ones close to 1500 nm (Figure 3H). The comparative result, observing the representative diagram of the appearance of histological sections focused on histiocytic sarcoma in Figure 3I, is based on Purzycka et al., 2020 [22]. This is where circular-shaped traces or spaces are observed in areas left without tissue or biological components (circular-shaped white spaces). This could be due to the accidental destruction and removal of PEVs during the processing of the samples themselves, as PEVs have a less stable or resistant structure than whole cells.

### 2.3. Confocal Microscopy

**Figure 4:**
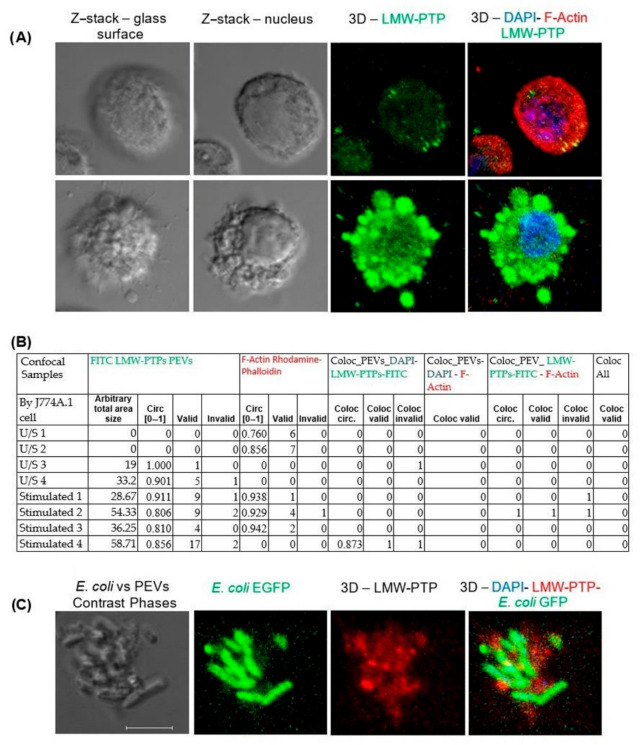
Confocal microscopy of J774A.1 PEVs and interaction with EGFP-positive *E. coli*. (**A**) J774A.1 cell lines were stimulated with SBMF-FN for 2 h, and PEVs were detected by their increased abundance of LMW-PTP protein (green). F-actin distribution was found in red, and nuclei stained with DAPI in blue. Images are 25 µm per side. (**B**) Detection of J774A.1 PEVs were analyzed with EV Analyzer imageJ2: Threshold 20, minimum circularity 0.5, method ‘Li’. The largest PEVs were only present in stimulated and unstimulated samples (U/S). (**C**) anti-LMW-PTP was used to identify J774A.1 PEVs (red—Alexa 594) that were placed in interaction with EGFP *E. coli* bacteria (green) and fixed after 5 min for the confocal microscopy procedure. Scale bar 5 µm.

Confocal microscopy confirmed the recognition of LMW-PTPs in J774A.1 PEVs. It was also identified that, in general, filamentous actin (F-Actin) is not observed in large PEVs (Figure 4A), with F-Actin being an important component of the cytoskeleton of cells. PEVs were corroborated in the analysis with EVAnalyzer, and the scarcity of PEVs with filamentous actin was confirmed. Focusing on the PEVs in contact with the glass surface containing the SBMF-FN substrate, we can see that the PEVs appear to be attached or anchored to the surface, possibly in an unstable manner, as they do not show F-actin or where the substrate was located (Figure 4A). When using PEVs and interacting them with EGFP bacteria—*E. coli* (fluorescent green) bacteria, we observe that J774A.1 cells are capable of adhering to the bacteria and even forming aggregates intercalated between bacteria—PEVs—bacteria (Figure 4C), a result that suggests that the PEVs adhered or anchored to the glass are so through the SBMF-FN substrate.

### 2.4. Phosphatase Activity of J774A.1PEVs

**Figure 5:**
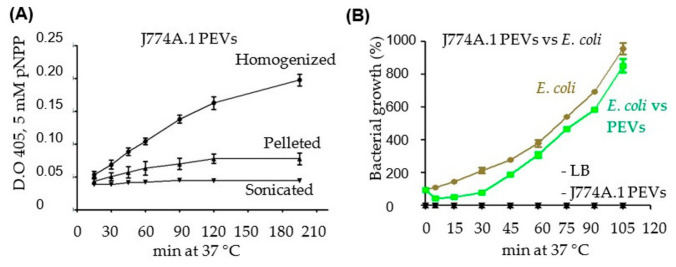
Biological phosphatase activity of J774A.1 PEVs with p-nitrophenylphosphatase (pNPP), and the effect of PEVs on bacterial growth. (**A**) J774A.1 PEVs from SBMF-FN stimulated cells were treated in three ways: vortexing the pellet (homogenized), placing the entire PEV pellet, or the PEV lysate by sonication. All PEVs were obtained from supernatants (without using ZnSO_4_), and the activity of 5 µg PEVs was measured for 15–195 min at 405 nm at 37 °C. (**B**) J774A.1 PEVs interacted with *E. coli* DH5α bacteria (green) by homogenization. As a positive control, only bacteria (brown) were used. As a negative control, LB medium alone and the LB PEVs mix were used (OD_600_; PEV density of 0.07 was subtracted as background from the entire system). The OD_600_ absorbance (0.070) of the bacteria, acquired at minute 0 of the start of the assay, was taken as 100%. *n* = 3.

By evaluating the phosphatase activity of PEVs, we also indirectly assessed whether these PEVs need to arrive “intact” at their target or target cells. By placing the PEVs in different conditions: one in which they were lysed by sonication, another in pellet form, and finally suspended (homogenized). We found that they exhibited the best phosphatase activity when they were “complete” and homogenized with the enzymatic reaction medium, followed by when they were tested in pellet form, and lastly, when they were sonicated (their phosphatase activity under this condition could be considered negligible), Figure 5A. The result also suggests that the method of preparation of the PEVs will significantly influence the biological activity to be analyzed and the accessibility of the phosphatase activity.

### 2.5. E. coli vs. J774A.1 PEVs Challenge

Using *E. coli* DH5α at a ratio of its OD_600_ to the OD_600_ of J774.1 PEVs, the samples were homogenized prior to the assay. Interactions were carried out at 37 °C. Figure 5B shows that between 5 and 15 min of PEVs-*E. coli* interaction, bacterial growth was considerably lower compared to the control bacteria in LB medium, a result that suggests an inhibitory and/or bactericidal activity of J774A.1 PEVs. After 45 min of evaluation, the bacteria showed a resumption of bacterial growth.

### 2.6. Analysis of Proteins Identified in J774A.1 PEVs by Mass Spectrometry

**Figure 6:**
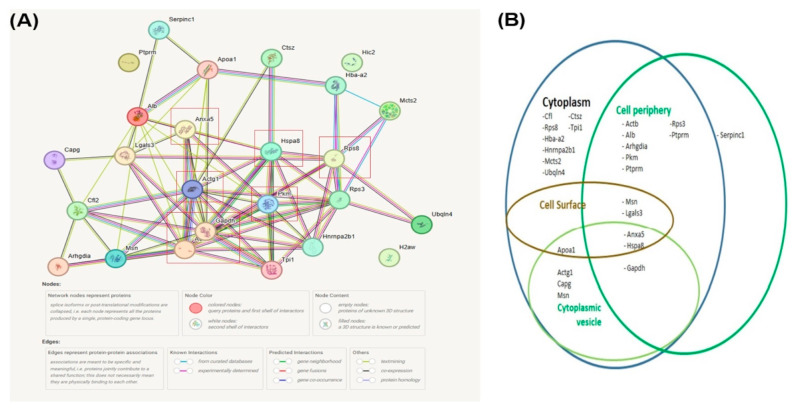
Protein–protein interaction network and Venn diagram of the main proteins identified in the J774A.1 PEVs secreted by stimulation with FN-SBMF. (**A**) The image shows the interactions, co-expressions, and homologies among the main proteins identified in the J774A.1 PEVs (Table S1). Red boxes indicate some nodes with multiple interactions within the network. (**B**) the main subcellular localization in the cytoplasm, cell periphery, cytoplasmic vesicle, and cell surface. Data were analyzed using STRING (version 12.0). Intersections indicate proteins with multiple or dynamic localization within the cell. Extensive descriptions are in Supplementary Figure S1 and Table S2.

Figure 6 presents an analysis of the main proteins identified in J774A.1 PEVs secreted after stimulation of cells with FN-SBMF. Figure 6A shows a protein–protein interaction network generated by STRING (version 12.0), illustrating the complex relationships between the 25 main proteins. In this network, significant direct and functional interactions are observed, including co-expression, protein associations, and homologies. Central nodes such as Gapdh, Tpi1, Pkm, Actg1, Hspa8, and Anxa5 stand out, acting as key points, suggesting that they play a fundamental role in the functionally coordinated structure of PEVs. The Venn diagram (Figure 6B) breaks down the common subcellular localization of these 25 proteins, focusing the analysis on four compartments: cell periphery, cytoplasmic vesicle and cell surface, and cytoplasm. Most proteins are located in the cytoplasm or cell periphery, with a significant group (including Anxa5, Hpsa8, Msn, Lgals3, and Gapdh) showing a shared location. In addition, the Anxa5 and Hspa8 proteins were found at an intersection of the four compartments evaluated, reflecting a dynamic and multiple localization within the cell, essential for their role in vesicular packaging and release.

## 3. Discussion

This study focused on the use of the J774A.1 cell line as a representative model for mammalian and human histiocytic sarcoma (HS) in their extracellular vesicle (EVs) secretory response. Currently, these cells have been used for the representative study of macrophages [8,9,10]; however, they secrete some proteins through polydisperse EVs (PEVs), which suggests similarities in terms of biomolecule transport activity to other types of cancer that have been studied [16,18,19]. The literature reports a basal release mainly of exosomes (30–150 nm) as part of physiological communication with other cells, with various targets, as well as in the manipulation of their microenvironment [14,19,20]. However, our results reveal that stimulation with PAMPs (Pathogen-Associated Molecular Patterns; *E. coli* bacterial membrane fraction containing OMPs, lipids, and LPS) dramatically increases the release of large and small extracellular vesicles, therefore PEVs, above basal levels, generating a hyper-induced secretory response.

This amplified release results in a population of PEVs, including a significant fraction of large vesicles that can be observed by confocal microscopy at close to 1–2 micrometers, classified in the literature as ‘Large Extracellular Vesicles’ (LEVs) or as large oncosomes when they contain properties exclusive to the cancer cells of origin [15,23,24,25]. The proteome of these J774A.1 PEVs, partially characterized by Sierra-López et al. 2025 [13] and determined by mass spectrometry in this work, confirm their functional nature and aggressive potential, which is key to understanding and developing HS. The literature includes histological images of HS [2,22,26,27,28] that we examined in detail. Based on these observations, we concluded that it is possible to identify footprint-like marks, cavities, or ghost-like circular or spherical structures at the periphery of histiocytic cancer cells. These features appear to persist after processing tissue samples of human or animal origin and, in our view, may correspond to large PEVs that were disintegrated during preparation. Notably, their appearance closely resembles the structures observed in our confocal and scanning electron microscopy analyses following stimulation of J774A.1 cells and their PEVs, which could explain why the circular void-like marks are reported in HS histological sections.

Proteomic analysis of induced PEVs reveals significant overlap with the molecular signatures of EVs reported in various types of cancer [17,18,29,30], underscoring the pathological nature of secretion. The high abundance of Actin (41.7 kDa) and Moesin (67.6 kDa), together with Cofilin (18.7 kDa), is characteristic of EVs that bud directly from the plasma membrane (microvesicles/ectosomes) [19,20,21,31]. These components are crucial for cell motility and invasion and are consistently found in exosomes and microvesicles from highly metastatic cancers such as melanoma and pancreatic cancer, where they promote the migration of recipient cells [32,33,34]. In our model, the enrichment of Moesin and Cofilin, driven by independent induction pathways, provides the molecular and structural basis for the cortical actin remodeling required to generate large vesicle blebbing. This specialized cargo not only architecturally defines the polydisperse nature of the secretome ‘PEVs’, but also actively equips these PEVs with the machinery needed to manipulate intercellular gaps in the microenvironment. The presence of Arhgdia (23.4 kDa), a regulator of the Rho actin pathway [35,36], reinforces the potential of these PEVs to modify the cytoskeleton and cell adhesion, a pro-migratory mechanism that we also observed phenotypically as a conserved translational response during the SBMF-stimulation of human U937 monocytes (Figure S2).

After the analysis of the mass spectrometry results, it is also possible to suggest a role for pro-aggressive and regulatory enzymes [23,37,38], among which the presence of Mu-type receptor tyrosine phosphatase (PTPRM) (163.6 kDa) and cathepsin was confirmed. The release of active PTPRM is a strategy employed by tumor cells and tumor-associated macrophages (TAMs) to deactivate crucial signaling pathways in lymphocytes [39], promoting immunosuppression and contributing to immune evasion in cancers [38]. On the other hand, cathepsin is a potent protease that contributes to the degradation of the extracellular matrix (ECM) and has been reported in EVs from lung and colorectal cancer, facilitating invasion [16,37,40]. It should be noted that the bacterial fraction was stabilized with the extracellular matrix component called fibronectin (FN). The strong proteolytic activity in total EVs extracts and the resulting degradation is another characteristic that has been documented in oncosomes and large oncosomes from multiple types of neoplasms [15,41]. In our case, we observed a strong susceptibility to degradation of (Low Molecular Weight Protein Tyrosine Phosphatases) LMW-PTPs in total extracts. LMW-PTPs have been detected and associated with the progression of chemotherapy resistance in multiple cancer types, such as colorectal, prostate, gastric, and leukemia [42].

Other proteins found in J774A.1 PEVs and identified in EVs from different neoplasms are those considered stress markers, such as Hsp71 (heat shock protein), Annexin A5 (35.7 kDa), and histone H2A, which suggests that the release of PEVs occurs under conditions of cellular stress (induced by PAMPs). This may also be related to the release of EVs that modulate apoptosis in target cells [11,31,43]. This is mainly supported by the fact that once the planned stimulation time was over, when medium and fetal bovine serum were added again, the previously stimulated J774A.1 line showed survival at 5 h into the assay.

The mechanism of EVs release upregulated by PAMPs, with its pro-aggressive molecular load, is crucial for understanding the pathobiology of HS (a macrophage neoplasm). The massive release of these enzyme-loaded oncosomes provides the tumor with an efficient mechanism for invasion and immunosuppression. Furthermore, the strong adhesion of these EVs to PAMPs indicates that the macrophage defense machinery can be hijacked by the sarcoma to promote adhesion to the ECM and metastatic colonization, a key factor in the aggressiveness of this type of cancer. Despite these significant findings, some limitations of the present study must be acknowledged, such as our reliance on a static in vitro model, the need to physically fractionate specific vesicle subpopulations to decipher their independent functional contributions and other human cell lines, and the characterization of downstream molecular targets of the identified phosphatases. However, these limitations further reinforce our hypothesis regarding the high utility of the J774A.1 cell line; as an immortalized model of histiocytic tumor origin (murine reticular cell sarcoma), it provides a direct, highly accessible, and reproducible analog that overcomes the yield restrictions of primary macrophages, establishing a robust and standardized platform to screen novel therapeutic targets in histiocytic sarcoma.

## 4. Materials and Methods

### 4.1. Extraction of Escherichia coli Fraction

Briefly, Gram-negative *E. coli* strain BL21 (DE3) pLysS bacteria were cultured in 300 mL of Luria-Bertani (LB) medium, and a soluble bacterial fraction was obtained as described in the method modified by Sierra-López et al. 2025 [13], in 15 mL of 10 mM Tris-HCl, pH 8.0, 10 mM MgCl_2_, and 2% SDS (fraction called SDS-SBMF). SDS-SBMF was dialyzed in a cold room (below 15 °C) using 15,000 Da membranes and 4 L of milliQ water per sample, ending dialysis after 72 h. The dialyzed SDS-SBMF was frozen until use.

### 4.2. Cell Culture

The J774A.1 murine macrophage/histiocytic sarcoma cell line (American Type Culture Collection, Manssas, VA, USA) from BALB/c mice was cultured in high-glucose-supplemented DMEM (Dulbecco’s Modified Eagle Medium), supplemented with 10% (*v*/*v*) heat-inactivated fetal bovine serum (FBS) (PAA, A15-701), 100 IU/mL of penicillin, and 100 µg/mL streptomycin. The cells were incubated at 37 °C in a humidified incubator with 5% CO_2_ and 95% air. The cells were FBS-starved prior to EV induction and assays.

### 4.3. J774A.1 PEVs (Short and Large EVs) Stimulation, Secretion, and Isolation

Briefly, glass slides and culture surfaces were coated with a mix of 0.250 µg of human fibronectin (FN) and 10–20 ng of dialyzed SDS-SBMF protein per cm^2^ as stimulus. The fraction called SBMF is mainly composed of the outer membrane fraction of *E. coli* bacteria, which includes porins and their various proteins (mainly OmpF, OmpA, OmpX, lpp), lipids, and suggests that it contains LPS. The protein profile of SBMF was obtained from mass spectrometry characterization as performed by Sierra-López et al. 2025 [13]. PEVs stimulation, secretion, and isolation were performed as indicated by Sierra-López et al. 2025 [13]. We added 4 × 10^4^ J774A.1 cells in DMEM (without serum) per cm^2^ of the culture surface with or without the stimulant, and were cultured for 120 min. The cells were fixed with 4% paraformaldehyde in PBS for 45 min for confocal microscopy assays, or processed for scanning electron microscopy, or PEVs in the supernatants were collected.

The J774A.1 PEVs were collected from the supernatant of stimulated cells in Petri dishes (diameter 10 cm) with 15 mL of DMEM (without serum) at around 50% confluence. Supernatants were first suspended via orbital movements (20/20s), and 14 mL were collected. Samples in 1.5 mL conical tubes (Eppendorf, Sait Louis, MO, USA) were centrifuged at 120× *g* for 5 min to sediment and remove cells together with 100 µL of adjacent supernatant. Then, 0.2 mM ZnSO_4_ was added to the supernatants to promote the association of large and short EVs, and the PEVs were pelleted via centrifugation at 12,000× *g* for 20 min (Centrifuge 5415C). ZnSO_4_ was not used for the PEVs vs. *E. coli* EGFP test.

### 4.4. Scanning Electron Microscopy (SEM)

J774A.1 PEVs/cells samples were fixed with paraformaldehyde after being permeabilized with 0.0015% SDS and 0.0006% Triton X-100 or 0.01% SDS and 0.004% Triton X-100 to decouple PEVs, performed as described by Sierra-López et al. 2025 [13]. The samples were washed with PBS, again fixed with 2.5% (*v*/*v*) glutaraldehyde in 0.1 M sodium cacodylate buffer, pH 7.2, and dehydrated with increasing concentrations of ethanol for SEM treatment. The samples were then critically point-dried with CO_2_ in a Samdri-780 Tousimis apparatus (Rockville, MD, USA). Afterward, in an ion-sputter device (Jeol-JFC-1100, Tokyo, Japan), they were gold-coated and examined with a Jeol JSM-7100F (Tokyo, Japan) field emission scanning electron microscope.

### 4.5. E. coli vs. J774A.1 PEVs Challenge

J774A.1 PEVs in LB (Luria Bertani) medium (310 µL) were placed in optical density (OD) cuvettes (4.5 × 1.2 × 1.0 cm) and cultured to an OD_600_ of 0.070–0.075. The PEVs were previously obtained (without ZnSO_4_) from 7 × 10^6^ cells stimulated with the SDS-SBMF bacterial fraction. Next, 310 µL of *E. coli* DH5α bacteria with an OD_600_ of 0.070 to 0.080 were added. LB medium alone was used as a control, and the OD value of LB medium with PEVs, with a volume that was completed to 620 µL, was subtracted from all readings containing PEVs.

For the samples to be analyzed by confocal microscopy, the interaction of PEVs and *E. coli* containing the pEGFP green fluorescent protein expression plasmid was performed. They were incubated on a glass slide containing the SDS-SBMF-FN stimulant for 5 min, in order to capture PEVs that could bind to both the substrate and the bacteria, and to analyze the time at which the least bacterial growth was observed in interaction with J774A.1 PEVs, which were analyzed by confocal microscopy.

### 4.6. Confocal Microscopy

J774A.1 cells were stimulated for 120 min with FN-SDS-SBMF on coverslips and fixed with 4% p-formaldehyde in PBS for 45 min at 37 °C. The samples were washed with PBS and permeabilized with 0.0015% SDS and 0.0006% Triton X-100 in PBS for 8 min at room temperature, and washed carefully with PBS once. The samples were blocked with 5% bovine serum and treated as described in Sierra-López et al. 2025 [13]. The cells and PEVs were incubated with either serum anti-LMW-PTPs (1:300) in PBS for 1 h, followed by anti-mouse FITC-conjugated secondary antibodies (Zymed Laboratories, South San Francisco, CA, USA; 1:300), or Rhodamine–Phalloidin (Sigma-Aldrich, St. Louis, MO, USA; 1:150) for 1 h at room temperature. Samples were preserved using Vectashield with DAPI Antifade Reagent (Vector, Newark, CA, USA), examined through a Carl Zeiss LMS 700 confocal microscope, and processed with ZEN 2010 (Zeiss, White Plains, NY, USA). The color green was used to represent LMW-PTP to analyze EVs with the ImageJ2 (version 2.16.0) EV Analyzer plugin (ImageJ/Fiji). To analyze the *E. coli* pEGFP vs. PEVs, these samples were fixed and treated with mouse serum anti-LMW-PTPs (1:300), Alexa 594 goat IgG anti-mouse IgG (Molecular Probes, Eugene, OR, USA; 1:300), and preserved with Vectashield (Vector, Newark, CA, USA).

### 4.7. Liquid Chromatography and MALDI-MS/MS

PEV protein identification was performed as described by Sierra-López et al. 2025 [13] from stimulated J774A.1 cells (with SDS-SBMF-FN substrate). The resulting MS/MS spectra were compared using Protein Pilot v.2.0.1 (ABSciex, Framingham, MA, USA) and the Paragon algorithm [44] against *Mus musculus* (Uniprot, 46,452 protein sequences database). The detection threshold was 1.3 to ensure 95% confidence. The identified proteins were grouped using the ProGroup algorithm to minimize redundancy. The proteins identified and used in this work are listed in Supplementary Table S1.

### 4.8. SDS-PAGE and Immunodot Blot

J774A.1 PEVs extracts and SDS-SBMF fraction were resolved on 12% SDS-PAGE and visualized via staining with Coomassie blue. The samples were treated with complete protease inhibitors and 0.1% paraformaldehyde to inhibit protease activity as described in Sierra-López et al. 2025 [13], and were mixed with loading buffer. Subsequently, the samples were placed in boiling water for 8 min for protein denaturation, and cooled on ice, followed by the addition of 8 µL of β-mercaptoethanol.

Immunodot blots were performed by placing 2 µL of each of the conditions of interest (suspended PEVs in supernatants of J774A.1 PEVS) and were incubated with mouse serum anti-LMW-PTP polyclonal antibody at a 1:1000 dilution. Bound antibodies were detected using alkaline phosphatase-conjugated goat anti-mouse IgG (H + L) (Invitrogen, Camarillo, CA, USA) at a 1:4000 dilution in TBST. Dot-blots were then developed with an NBT/BCIP substrate kit (Invitrogen, Camarillo, CA, USA).

### 4.9. Phosphatase Activity of J774A.1 PEVs

The phosphatase activity of PEVs without ZnSO_4_ was analyzed by hydrolysis of the substrate p-nitrophenyl phosphate (pNPP) (Sigma) in 96-well plates. For each assay, 100 µL of enzyme reaction with pNPP, a mixture was first prepared with 60 µL of 200 mM sodium acetate, pH 6.0, 6.25 µL of 100 mM dithiothreitol (DTT), 5 µg of PEVs, and milliQ water was added to a total volume of 90 µL. The mixture was incubated for 10 min at room temperature, then 2.5 µL of 100 mM pNPP was added and mixed, and incubated at 37 °C. The activity was revealed by adding 10 µL of 2 M NaOH at the designated times (10, 15, 20, 40, 80, 120, 140 min). The absorbance was read at 405 nm in 96-well plates.

### 4.10. Statistical Analysis

Confocal analysis of EV secretion by macrophages was performed using Fiji and the EV Analyzer plugin (version 8.1.3 beta). Function: EV coloc; minimum threshold: 20; threshold method: ‘Li’; min circularity: 0.5; filter type: EV-GFP (to FITC channel); particle size range: 1–999,999. In unstimulated macrophages, cells that secrete EVs were sought and analyzed (<20% cells). The EV Analyzer data description was analyzed using GraphPad Prism 5, one-way ANOVA analysis of variance, and Bonferroni’s multiple comparison post-test. Protein–protein interaction network and Venn diagram data of the main proteins identified in the J774A.1 PEVs secreted by stimulation with FN-SBMF were obtained using STRING (version 12.0).

## 5. Conclusions

The J774A.1 cell line can be considered a suitable model for studying the secretion of polydisperse HS EVs, given that this cell line was originally derived from ascites tumors in a mouse with reticular cell sarcoma, a previous classification that encompasses neoplasms of the macrophage lineage, among others. It has also been used in vitro as a model for studying macrophages and has the characteristic of being immortalized cells that proliferate, a set of characteristics possessed by histiocytic sarcoma cells. Stimulation with *E. coli* SBMF has dramatically amplified the release of PEVs (including those similar to oncosomes and large oncosomes) above the basal secretion, which is known to be dominated by exosomes in the J774A.1 cell line. The morphology of J774A.1 cells, when stimulated with *E. coli* SBMF and stabilized with FN, resemble the appearance of HS neoplastic cells in reported histological sections, where large PEVs were apparently destroyed by the treatment of the samples, leaving traces or ghosts of them marked in the histological sections. These PEVs are multifunctional units whose proteomic load includes factors associated with invasion in other types of cancer (e.g., actin, moesin, cathepsin) and the active phosphatase PTPRM. This hyper-induced release mechanism, which integrates the immune response with the capacity for invasion, positions J774A.1 PEVs as key pathological participants and a therapeutic target with great potential in HS, making them candidates for study in conjunction with or separately from a possible approach involving oncosomes and large oncosomes.

## Data Availability

The original contributions presented in this study are included in the article/Supplementary Material. Further inquiries can be directed to the corresponding authors.

## Supplementary Materials

The following supporting information can be downloaded at: https://www.mdpi.com/article/10.3390/ijms27114949/s1.

## Abbreviations

The following abbreviations are used in this manuscript:

EVs	Extracellular Vesicles
ECM	Extracellular matrix
EGFP	Enhance green fluorescent protein
F-Actin	Filamentous actin
FN	Fibronectin
SH	sarcoma Histiocytic
LB	Luria-Bertani culture medium
MS	mass spectrometer
MW	molecular weight marker
PEVs	Polydispersity extracellular vesicle
pNPP	substrate p-nitrophenyl phosphate
SBMF	fraction of the outer membrane of Escherichia coli bacteria
SEM	Scanning Electron Microscopy
TAMs	Tumor-associated macrophages
TPI	Triosephosphate isomerase
