# Supplementary material for: Use of the J774A.1 Cell Line as a Model in the In Vitro Study of Extracellular Vesicle Secretion from Histiocytic Sarcoma in Patients with Bacterial Co-Infections
Source: Int J Mol Sci. 2026 May 29;27(11):4949. doi: 10.3390/ijms27114949 (PMC13257283; doi:10.3390/ijms27114949)
Supplement: Supplementary file 1 [file ijms-27-04949-s001.zip › ijms-4269440-supplementary.pdf]

Table S1. The protein profile of the J774A.1 PEVs is reported in Sierra-López et al 2025. Additionally, in this study, a minimum cutoff value of 'Unused' (U) of 1.9 was used, and the total number of identified proteins were listed from highest 'U'. A1-A7 corresponds to the approximate area of the SDS-PAGE gel homologous to the SDS-PAGE from which they were originally identified.

Entry: Mouse	U	Name: OS=Mus musculus	SDS-PAGE Zone	Subcellular Localization 'Compartments' (STRING): Cytoplasm, Cell periphery, Cytoplasmic vesicle, Cell surface.
P07724 ALBU	18.06	Serum albumin GN=Alb PE=1 SV=3	A7	Cytoplasm, Cell periphery.
P17751 TPIS	12	Triosephosphate isomerase GN=Tpi1 PE=1 SV=4	A4	Cytoplasm
P63017 HSP7C	10.89	Heat shock cognate 71 kDa protein GN=Hspa8 PE=1 SV=1	A2	Cytoplasm, Cell periphery, Cytoplasmic vesicle, Cell surface
Q00623 APOA1	10.66	Apolipoprotein A-I GN=Apoa1 PE=1 SV=2	A4	Cytoplasm, Cytoplasmic vesicle, Cell surface
Q99PT1 GDIR1	6.03	Rho GDP-dissociation inhibitor 1 GN=Arhgdia PE=1 SV=3	A4	Cytoplasm, Cell periphery
A0A0A0MQF6 Gapdh	6.02	Glyceraldehyde-3-phosphate dehydrogenase GN=Gapdh PE=1 SV=1	A4	Cytoplasm, Cell periphery, Cytoplasmic vesicle
Q8C253 Galectin	6	Galectin GN=Lgals3 PE=1 SV=1	A4	Cytoplasm, Cell periphery, Cell surface
P07724 ALBU	4	Serum albumin GN=Alb PE=1 SV=3	A1	Cytoplasm, Cell periphery
P26041 MOES	2.66	Moesin GN=Msn PE=1 SV=3	A2	Cytoplasm, Cell periphery, Cytoplasmic vesicle Cell surface
P63260 ACTG	2.45	Actin, cytoplasmic 2 GN=Actg1 PE=1 SV=1	A3	Cytoplasm
P07724 ALBU	2.02	Serum albumin GN=Alb PE=1 SV=3	A2	Cytoplasm, Cell periphery
P48036 ANXA5	2.02	Annexin A5 GN=Anxa5 PE=1 SV=1	A4	Cytoplasm, Cell periphery, Cytoplasmic vesicle, Cell surface
P02089 HBB2	2.02	Hemoglobin subunit beta-2 GN=Hbb-b2 PE=1 SV=2	A4	Cytoplasm
P28828 PTPRM	2	Receptor-type tyrosine-protein phosphatase mu GN=Ptprm PE=2 SV=2	A1	Cell periphery
Q9CQ21 MCTS2	2	Malignant T-cell-amplified sequence 2 GN=Mcts2 PE=2 SV=1	A1	Cytoplasm
Q99NB8 UBQL4	2	Ubiquilin-4 GN=Ubqln4 PE=1 SV=1	A1	Cytoplasm,
P52480 KPYM	2	Pyruvate kinase PKM GN=Pkm PE=1 SV=4	A2	Cytoplasm, Cell periphery
P62242 RS8	2	40S ribosomal protein S8 GN=Rps8 PE=1 SV=2	A4	Cytoplasm
Q8BFU2 H2A3	2	Histone H2A type 3 GN=Hist3h2a PE=1 SV=3	A4	(Nucleus, EVpedia: EVs)*
Q9JLZ6 HIC2	2	Hypermethylated in cancer 2 protein GN=Hic2 PE=2 SV=4	A4	(Nucleus, EVpedia: EVs)*

P45591 COF2	2	Cofilin-2 GN=Cfl2 PE=1 SV=1	A4	Cytoplasm
J3QK04 MCG67952	2	MCG67952 GN=Gm7808 PE=4 SV=1	A4	Cytoplasm, EVs
E9Q5F4 Actb	2	Actin, cytoplasmic 1 (Fragment) GN=Actb PE=1 SV=1	A4	Cytoplasm, Cell periphery
Q9WUU7 CATZ	2	Cathepsin Z GN=Ctsz PE=2 SV=1	A4	Cytoplasm
P62908 RS3	2	40S ribosomal protein S3 GN=Rps3 PE=1 SV=1	A4	Cytoplasm, Cell periphery
Q91VB8 Q91VB8	2	Alpha globin 1 GN=hemoglobin alpha 2 PE=1 SV=1. Hba-a2	A4	Cytoplasm
O88569 ROA2	2	Heterogeneous nuclear ribonucleoproteins A2/B1 GN=Hnrnpa2b1 PE=1 SV=2	A4	Cytoplasm
P63260 ACTG	2	Actin, cytoplasmic 2 GN=Actg1 PE=1 SV=1	A7	Cytoplasm, Cytoplasmic vesicle
P32261 ANT3	2	Antithrombin-III GN=Serpinc1 PE=1 SV=1	A7	Cell periphery
Q99LB4 Capg	1.96	Capping protein (Actin filament), gelsolin-like GN=Capg PE=1 SV=1	A3	Cytoplasm, Cytoplasmic vesicle

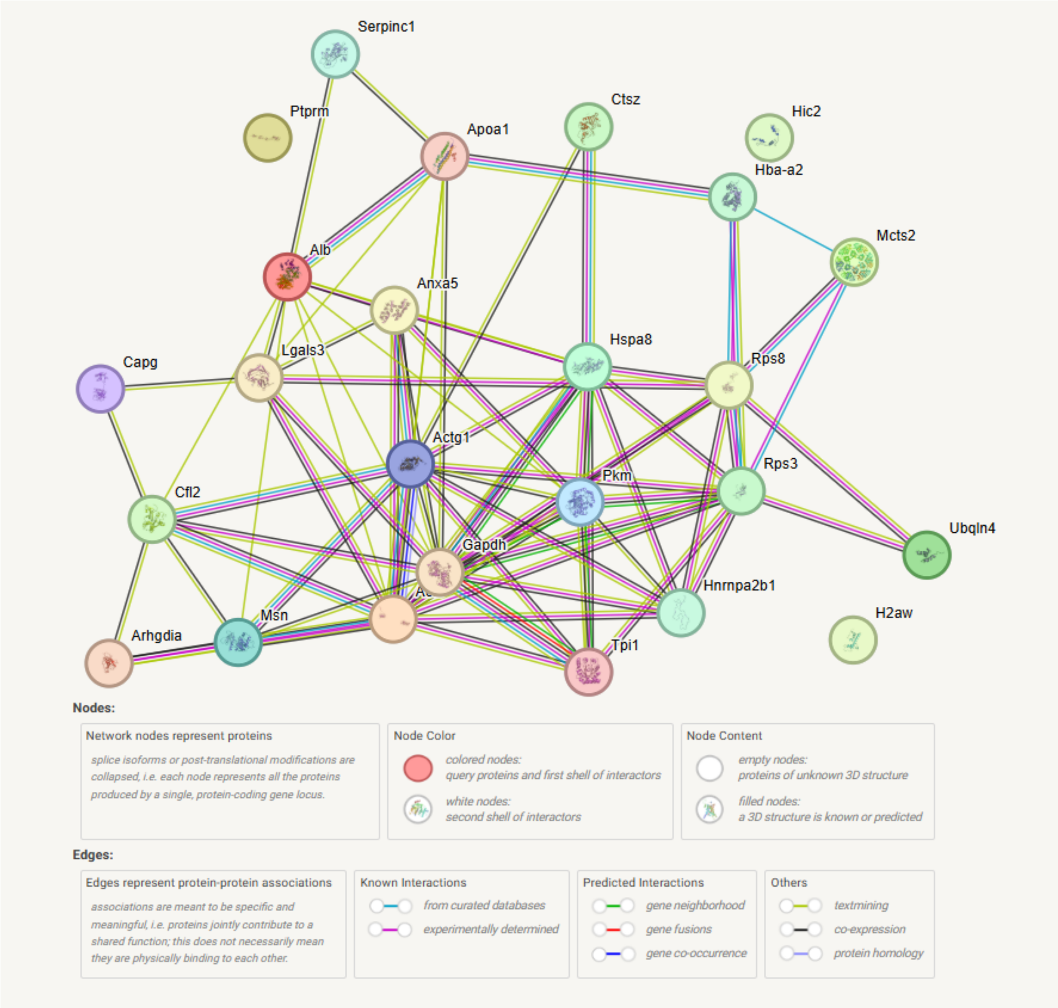

Figure S1. Protein-Protein Interaction Network (Interactome) of Polydisperse Extracellular Vesicle (PEVs) Contents Released by J774A.1 Cells. This figure represents the interactome of proteins identified by mass spectrometry in an extract of polydisperse extracellular vesicles. These vesicles were secreted by the J774A.1 cell line, which was stimulated for two hours with SDS-SBMF *E. coli* stabilized with FN. The analysis of the proteins identified by mass spectrometry in the PEVs was performed using the STRING server (version 12.0). Nodes represent proteins (red: query proteins and first interactor layer; white: second interactor layer; filled circles: known 3D structure), and edges represent the associations between them. Evidence of interactions is color-coded: known interactions are derived from curated databases (light blue) or were experimentally determined (purple), while predicted interactions include gene proximity (green), gene fusions (red), and gene co-occurrence (dark blue). Other associations are derived from text mining (yellow/light green), co-expression (black), or protein homology (gray). This interactome illustrates how stimulation of J774A.1 cells (a model histiocytic sarcoma) with E. coli SBMF modulates the protein load of released PEVs, suggesting potential intercellular communication pathways in the context of inflammation and cancer. The main proteins represented in the network include ActB (Beta-actin), Actg1 (Gamma-actin 1), Gapdh (Glyceraldehyde-3-phosphate dehydrogenase), Hspa8 (71 kDa cognate heat shock protein), ANXA5 (Annexin A5), Lgals3 (Galectin-3), Capg (Actin filament capping protein, gelsolin-like), Hnrnpa2b1 (Heterogeneous nuclear ribonucleoprotein A2/B1), Msn (Moesin), Pkm (Pyruvate kinase, muscle), TPI1 (Triose phosphate isomerase 1), H2aw (Histone H2A), RPS3 (Ribosomal protein S3), Cfl2 (Cofilin 2), Ubqln4 (Ubiquilin 4), Hba-a2 (Hemoglobin alpha-2), Serpinc1 (Inhibitor of serpins (clade C, member 1), Apoa1 (Apolipoprotein A-I), Ptprm (Protein tyrosine phosphatase, mu-type receptor), Mcts2 (MCTS family member 2), and Arhgdia (Rho GDP dissociation inhibitor alpha). The interactome of the PEVs produced in abundance after SBMF-FN stimulation suggests that induction promotes expression in histiocytic sarcoma cells in ‘highly communicative and highly aggressive cells’ by releasing PEVs rich in survival and migration machinery. The result shows a molecular connection between microenvironmental/inflammatory stress (triggered by the SBMF E. coli), the tumor niche (FN, fibronectin), and the progression/aggressiveness (Actins/HSPs) of the histiocytic sarcoma, suggesting that the inhibition of the release or delivery of the cargo in the J774A.1 PEVs could be a target of a therapeutic strategy against this very aggressive cancer, one of them focused on the inhibition of catalytically active proteins present in the PEVs, such as possibly the HSPs found.

Table S2. Proteins identified by mass spectrometry, of the J774A.1 PEVs that were released by induction with SBFM-FN and recognized by STRING server (version 12.0, https://string-db.org/). *Mus musculus* NCBI taxonomy Id: 10090.

Alb	*Serum albumin; Serum albumin, the main protein of plasma, has a good binding capacity for water, Ca(2+), Na(+), K(+), fatty acids, hormones, bilirubin and drugs. Its main function is the regulation of the colloidal osmotic pressure of blood. Major zinc transporter in plasma, typically binds about 80% of all plasma zinc (By similarity). Major calcium and magnesium transporter in plasma, binds approximately 45% of circulating calcium and magnesium in plasma (By similarity). Potentially has more than two calcium-binding sites and might additionally bind calcium in a non-specific manner (B [...] (608 aa)*
Actb	*Actin, cytoplasmic 1, N-terminally processed; Actin is a highly conserved protein that polymerizes to produce filaments that form cross-linked networks in the cytoplasm of cells (By similarity). Actin exists in both monomeric (G-actin) and polymeric (F-actin) forms, both forms playing key functions, such as cell motility and contraction (By similarity). In addition to their role in the cytoplasmic cytoskeleton, G- and F-actin also localize in the nucleus, and regulate gene transcription and motility and repair of damaged DNA. (375 aa)*
Ptprm	*Receptor-type tyrosine-protein phosphatase mu; Involved in cell-cell adhesion through homophilic interactions. May play a key role in signal transduction and growth control. (1452 aa)*
Mcts2	*Malignant T-cell-amplified sequence 2. (181 aa)*
Ubqln4	*Ubiquilin-4; Regulator of protein degradation that mediates the proteasomal targeting of misfolded, mislocalized or accumulated proteins (By similarity). Acts by binding polyubiquitin chains of target proteins via its UBA domain and by interacting with subunits of the proteasome via its ubiquitin-like domain (By similarity). Key regulator of DNA repair that represses homologous recombination repair: in response to DNA damage, recruited to sites of DNA damage following phosphorylation by ATM and acts by binding and removing ubiquitinated MRE11 from damaged chromatin, leading to MRE11 de [...] (596 aa)*
Hspa8	*Heat shock cognate 71 kDa protein; Molecular chaperone implicated in a wide variety of cellular processes, including protection of the proteome from stress, folding and transport of newly synthesized polypeptides, activation of proteolysis of misfolded proteins and the formation and dissociation of protein complexes. Plays a pivotal role in the protein quality control system, ensuring the correct folding of proteins, the re-folding of misfolded proteins and controlling the targeting of proteins for subsequent degradation. This is achieved through cycles of ATP binding, ATP hydrolysis a [...] (646 aa)*
Msn	*Moesin; Ezrin-radixin-moesin (ERM) family protein that connects the actin cytoskeleton to the plasma membrane and thereby regulates the structure and function of specific domains of the cell cortex. Tethers actin filaments by oscillating between a resting and an activated state providing*

	transient interactions between moesin and the actin cytoskeleton (By similarity). Once phosphorylated on its C-terminal threonine, moesin is activated leading to interaction with F-actin and cytoskeletal rearrangement (By similarity). These rearrangements regulate many cellular processes, including cel [...] (577 aa)
Pkm	Pyruvate kinase PKM; Glycolytic enzyme that catalyzes the transfer of a phosphoryl group from phosphoenolpyruvate (PEP) to ADP, generating ATP (By similarity). Stimulates POU5F1-mediated transcriptional activation (By similarity). Promotes in a STAT1-dependent manner, the expression of the immune checkpoint protein CD274 in ARNTL/BMAL1-deficient macrophages. (531 aa)
Actg1	Actin, cytoplasmic 2, N-terminally processed; Actins are highly conserved proteins that are involved in various types of cell motility and are ubiquitously expressed in all eukaryotic cells. (375 aa)
Capg	Macrophage-capping protein; Calcium-sensitive protein which reversibly blocks the barbed ends of actin filaments but does not sever preformed actin filaments. May play an important role in macrophage function. May play a role in regulating cytoplasmic and/or nuclear structures through potential interactions with actin. May bind DNA. Uncapping occurs either when Ca(2+) falls or when the concentration of polyphosphoinositide rises, both at low and high Ca(2+); Belongs to the villin/gelsolin family. (349 aa)
Tpi1	Triosephosphate isomerase; Triosephosphate isomerase is an extremely efficient metabolic enzyme that catalyzes the interconversion between dihydroxyacetone phosphate (DHAP) and D-glyceraldehyde-3-phosphate (G3P) in glycolysis and gluconeogenesis. (299 aa)
Apoa1	Truncated apolipoprotein A-I; Participates in the reverse transport of cholesterol from tissues to the liver for excretion by promoting cholesterol efflux from tissues and by acting as a cofactor for the lecithin cholesterol acyltransferase (LCAT). As part of the SPAP complex, activates spermatozoa motility; Belongs to the apolipoprotein A1/A4/E family. (264 aa)
Arhgdia	Rho GDP-dissociation inhibitor 1; Controls Rho proteins homeostasis. Regulates the GDP/GTP exchange reaction of the Rho proteins by inhibiting the dissociation of GDP from them, and the subsequent binding of GTP to them. Retains Rho proteins such as CDC42, RAC1 and RHOA in an inactive cytosolic pool, regulating their stability and protecting them from degradation. Actively involved in the recycling and distribution of activated Rho GTPases in the cell, mediates extraction from membranes of both inactive and activated molecules due its exceptionally high affinity for prenylated forms. T [...] (204 aa)
Gapdh	Glyceraldehyde-3-phosphate dehydrogenase; Has both glyceraldehyde-3-phosphate dehydrogenase and nitrosylase activities, thereby playing a role in glycolysis and nuclear functions, respectively. Glyceraldehyde-3-phosphate dehydrogenase is a key enzyme in glycolysis that catalyzes the first step of the pathway by converting D-glyceraldehyde 3-phosphate (G3P) into 3-phospho-D-glyceroyl phosphate. Modulates the organization and assembly of the cytoskeleton. Facilitates the CHP1-dependent microtubule and membrane associations through its ability to stimulate the binding of CHP1 to microtubu [...] (359 aa)
Lgals3	Galectin-3; Galactose-specific lectin which binds IgE. May mediate with the alpha-3, beta-1 integrin the stimulation by CSPG4 of endothelial cells migration. Together with DMBT1, required for terminal differentiation of columnar epithelial cells during early embryogenesis. In the nucleus: acts as a pre-mRNA splicing factor. Involved in acute inflammatory responses including neutrophil activation and adhesion, chemoattraction of monocytes macrophages, opsonization of apoptotic

	neutrophils, and activation of mast cells. Together with TRIM16, coordinates the recognition of membrane damage [...] (264 aa)
Anxa5	*Annexin A5; This protein is an anticoagulant protein that acts as an indirect inhibitor of the thromboplastin-specific complex, which is involved in the blood coagulation cascade; Belongs to the annexin family. (319 aa)*
Rps8	*40S ribosomal protein S8; Belongs to the eukaryotic ribosomal protein eS8 family. (208 aa)*
H2aw	*Histone H2A type 3; Core component of nucleosome. Nucleosomes wrap and compact DNA into chromatin, limiting DNA accessibility to the cellular machineries which require DNA as a template. Histones thereby play a central role in transcription regulation, DNA repair, DNA replication and chromosomal stability. DNA accessibility is regulated via a complex set of post-translational modifications of histones, also called histone code, and nucleosome remodeling. (130 aa)*
Hic2	*Hypermethylated in cancer 2 protein; Transcriptional repressor; Belongs to the krueppel C2H2-type zinc-finger protein family. Hic subfamily. (619 aa)*
Cfl2	*Cofilin-2; Controls reversibly actin polymerization and depolymerization in a pH-sensitive manner. It has the ability to bind G- and F-actin in a 1:1 ratio of cofilin to actin. It is the major component of intranuclear and cytoplasmic actin rods. Required for muscle maintenance. May play a role during the exchange of alpha-actin forms during the early postnatal remodeling of the sarcomere. (166 aa)*
Ctsz	*Cathepsin Z; Exhibits carboxy-monopeptidase as well as carboxy-dipeptidase activity (By similarity). Capable of producing kinin potentiating peptides (By similarity). (306 aa)*
Rps3	*40S ribosomal protein S3; Involved in translation as a component of the 40S small ribosomal subunit (By similarity). Has endonuclease activity and plays a role in repair of damaged DNA. Cleaves phosphodiester bonds of DNAs containing altered bases with broad specificity and cleaves supercoiled DNA more efficiently than relaxed DNA (By similarity). Displays high binding affinity for 7,8-dihydro-8- oxoguanine (8-oxoG), a common DNA lesion caused by reactive oxygen species (ROS) (By similarity). Has also been shown to bind with similar affinity to intact and damaged DNA (By similarity). S [...] (243 aa)*
Hba-a2	*Hemoglobin subunit alpha; Involved in oxygen transport from the lung to the various peripheral tissues; Belongs to the globin family. (142 aa)*
Hnrnpa2b1	*Heterogeneous nuclear ribonucleoproteins A2/B1; Heterogeneous nuclear ribonucleoprotein (hnRNP) that associates with nascent pre-mRNAs, packaging them into hnRNP particles. The hnRNP particle arrangement on nascent hnRNA is non-random and sequence-dependent and serves to condense and stabilize the transcripts and minimize tangling and knotting. Packaging plays a role in various processes such as transcription, pre-mRNA processing, RNA nuclear export, subcellular location, mRNA translation and stability of mature mRNAs. Forms hnRNP particles with at least 20 other different hnRNP and he [...] (341 aa)*
Serpinc1	*Antithrombin-III; Most important serine protease inhibitor in plasma that regulates the blood coagulation cascade. AT-III inhibits thrombin, matriptase-3/TMPRSS7, as well as factors IXa, Xa and XIa. Its inhibitory activity is greatly enhanced in the presence of heparin (By similarity). (465 aa)*

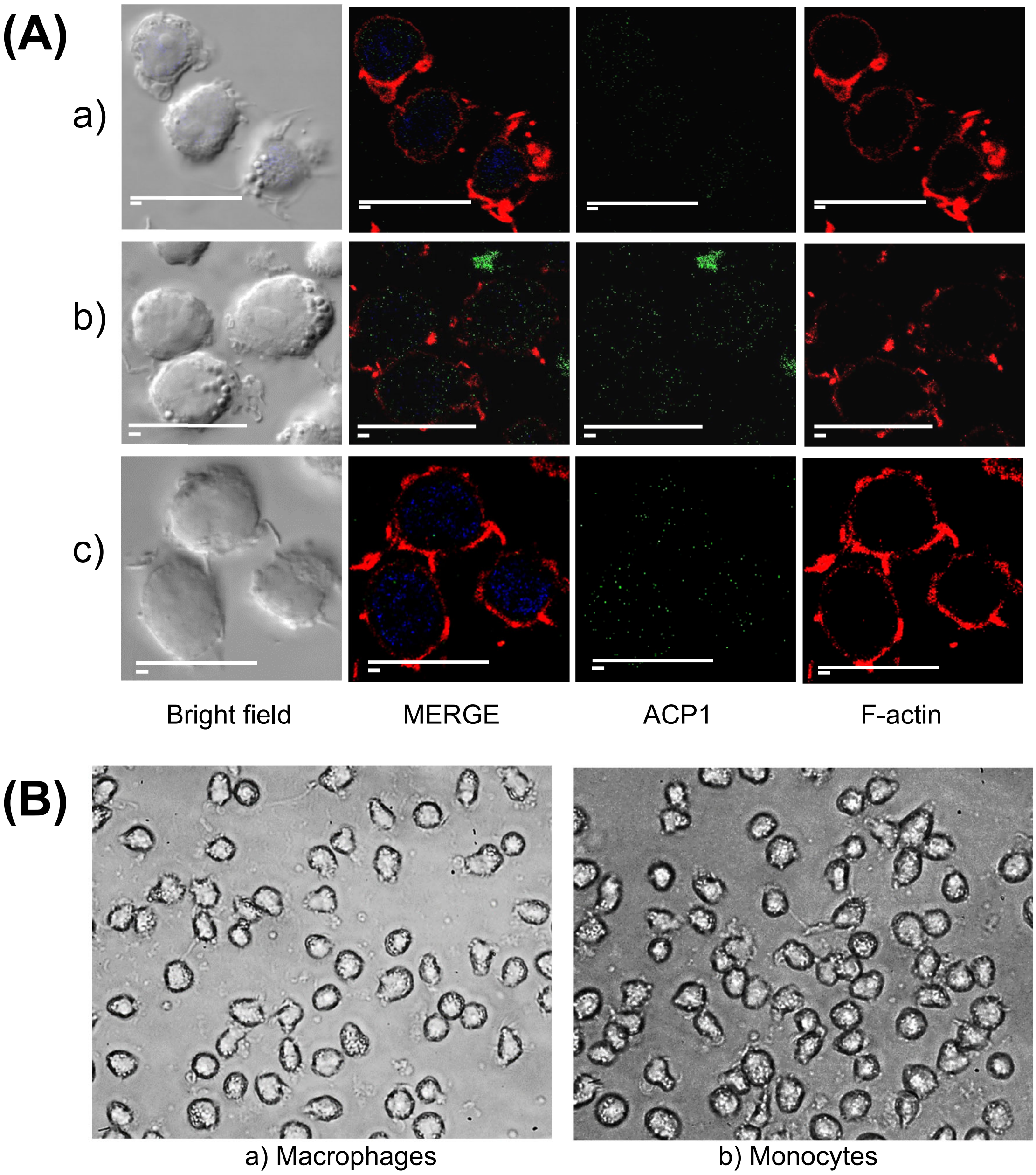

Figure S2. The SBMF fraction of *E. coli* induces U937 monocytes to secrete PEVs. (A) Monocytes were cultured for 4 hrs at 37 °C on SBMF + FN film (a-b) or without stimulation (c). a) Monocytes secreting large PEVs. b) Some membrane extensions were labeled with anti-LMW-PTP (green), while most monocytic VEPs were not (suggesting different markers between macrophage and monocyte PEVs). Rhodamine-phalloidin detected F-actin (red), and DAPI nucleolus (blue). Representative images from 5 assays. Scale bar: 20 and 2 µm. (B) Representative images of cells cultured at 37 °C on SBMF + FN film. Cells were observed under a light microscope (20x objective). a) Macrophages secreting large PEVs at 2 h. b) Monocytes secreting large PEVs at 4 h. Human monocytic cells line U937 (American Type Culture Collection, Rockville, MD, USA) were cultured in RPMI 1640

medium (with 2.05 mM L-glutamine) (HyClone, Logan, UT, USA) supplemented with 10% bovine serum (BS) (previously heat-inactivated), at 37 °C, 95% air and 5% CO2.

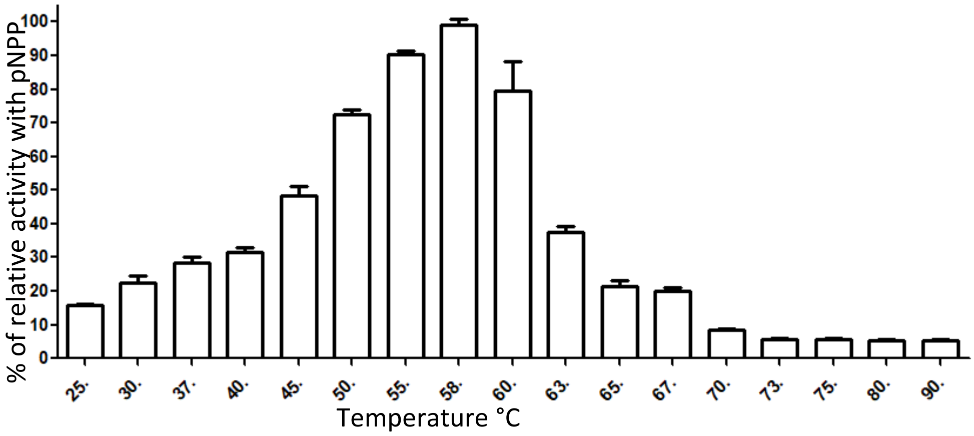

Figure S3. Effect of temperature on the activity of recombinant HsLMW-PTP protein. Catalytic activity was measured at different temperatures, as shown after 10 min of incubation in a reaction solution of 2 mM pNPP, 100 mM sodium acetate, and 5 mM DTT (dithiothreitol) in a volume of 100 microliters, which was stopped with 10 microliters of 2 M NaOH. The average of the maximum absorbance at 405 nm after 10 min of reaction was selected and assigned 100% activity. The representative assay was performed in triplicate, and the standard deviation is shown. Recombinant human HsLMW-PTP (GenBank sequence NP_009030 - HsLMW-PTP-b, HsACP1b) was obtained from Sierra-López et al. 2025.
